# Improvement of invasive microvascular indices following guideline-directed treatment of structural coronary microvascular dysfunction: a case report

**DOI:** 10.1093/ehjcr/ytag533

**Published:** 2026-07-14

**Authors:** Jérémie Buri, Aurelia Zimmerli, Bernard De Bruyne, Thabo Mahendiran, Stephane Fournier

**Affiliations:** Department of Cardiology, Lausanne University Hospital (CHUV), Rue du Bugnon 46, CH-1011 Lausanne, Switzerland; Department of Cardiology, Lausanne University Hospital (CHUV), Rue du Bugnon 46, CH-1011 Lausanne, Switzerland; Department of Cardiology, Lausanne University Hospital (CHUV), Rue du Bugnon 46, CH-1011 Lausanne, Switzerland; Cardiovascular Center Aalst, AZORG, Moorselbaan 164, B-9300 Aalst, Belgium; Department of Cardiology, Lausanne University Hospital (CHUV), Rue du Bugnon 46, CH-1011 Lausanne, Switzerland; Department of Cardiology, Lausanne University Hospital (CHUV), Rue du Bugnon 46, CH-1011 Lausanne, Switzerland

**Keywords:** Coronary microvascular dysfunction, INOCA, Continuous thermodilution, CFR, Microvascular resistance, Medical therapy, Case report

## Abstract

**Background:**

Structural coronary microvascular dysfunction (CMD) is a major pathophysiological mechanism underlying ischaemia with non-obstructive coronary arteries and is associated with persistent angina and impaired functional capacity. Although contemporary European guidelines recommend a structured, stepwise pharmacological approach, invasive confirmation of physiological improvement following this specific treatment protocol remains limited.

**Case summary:**

An 82-year-old woman presented with typical angina and stress-induced ischaemia on myocardial scintigraphy. Coronary angiography demonstrated a proximal left anterior descending artery stenosis <50% with a fractional flow reserve (FFR) of 0.90. Continuous thermodilution performed in the left anterior descending artery revealed reduced coronary flow reserve (CFR 2.2) and elevated minimal microvascular resistance (798 WU), consistent with structural CMD.

A structured, symptom-guided pharmacological optimization strategy was initiated, including statin therapy, angiotensin-converting enzyme inhibition, beta-blockade, calcium-channel blockade, and ranolazine.

At 4-month follow-up, angina was markedly improved. Repeat invasive assessment in the same coronary territory demonstrated improvement of microvascular indices (CFR 3.8; minimal microvascular resistance 461 WU).

**Discussion:**

This case illustrates that guideline-directed medical therapy for coronary microvascular dysfunction may be associated with both symptomatic improvement and objective changes in microvascular function.

Learning points

- Guideline-directed medical therapy in structural coronary microvascular dysfunction may be associated with objective improvement in invasive physiological indices.
- Continuous thermodilution enables direct quantification of coronary flow and microvascular resistance and is suitable for longitudinal intra-patient assessment.
- Discordance between bolus and continuous thermodilution measurements may occur in INOCA patients and requires integrated interpretation within the overall clinical and physiological context.

## Introduction

Ischaemia with non-obstructive coronary arteries (INOCA) is an increasingly recognized cause of angina and reduced quality of life.^1^ Among the underlying mechanisms, coronary microvascular dysfunction (CMD) represents a major pathophysiological endotype.^2^

Structural CMD reflects impaired hyperaemic vasodilatory capacity and is typically identified by reduced coronary flow reserve (CFR <2.5) in conjunction with increased minimal microvascular resistance >500 Wood units (WU) on invasive assessment. Coronary microvascular function can be evaluated invasively using both bolus and continuous thermodilution. Whilst bolus thermodilution permits the measurement of the CFR and the index of microcirculatory resistance (IMR), a surrogate for minimal microvascular resistance, continuous thermodilution permits the direct quantification of absolute coronary blood flow and microvascular resistance.^3–5^ Continuous thermodilution demonstrates high reproducibility, has been validated against positron emission tomography for absolute flow quantification, and enables direct assessment of coronary blood flow and minimal microvascular resistance. These characteristics make it particularly suited for longitudinal intra-patient follow-up when identical coronary territories and infusion protocols are used.^4–8^

Contemporary European guidelines and European Association of Percutaneous Cardiovascular Interventions (EAPCI) consensus documents recommend a structured, stepwise pharmacological approach targeting endothelial function, vascular tone, and myocardial oxygen demand in symptomatic INOCA patients.^1,9^ However, although stratified medical therapy has been shown to improve symptoms in randomized settings such as the CorMicA trial, invasive documentation of changes in microvascular function during follow-up remains scarce, in particular with the specific medical protocol proposed in the EAPCI expert consensus.^10^

We report a case of structural CMD associated with improvement of invasively assessed microvascular function following sequential guideline-directed therapy within a structured INOCA management framework.

## Summary figure

**Figure:**
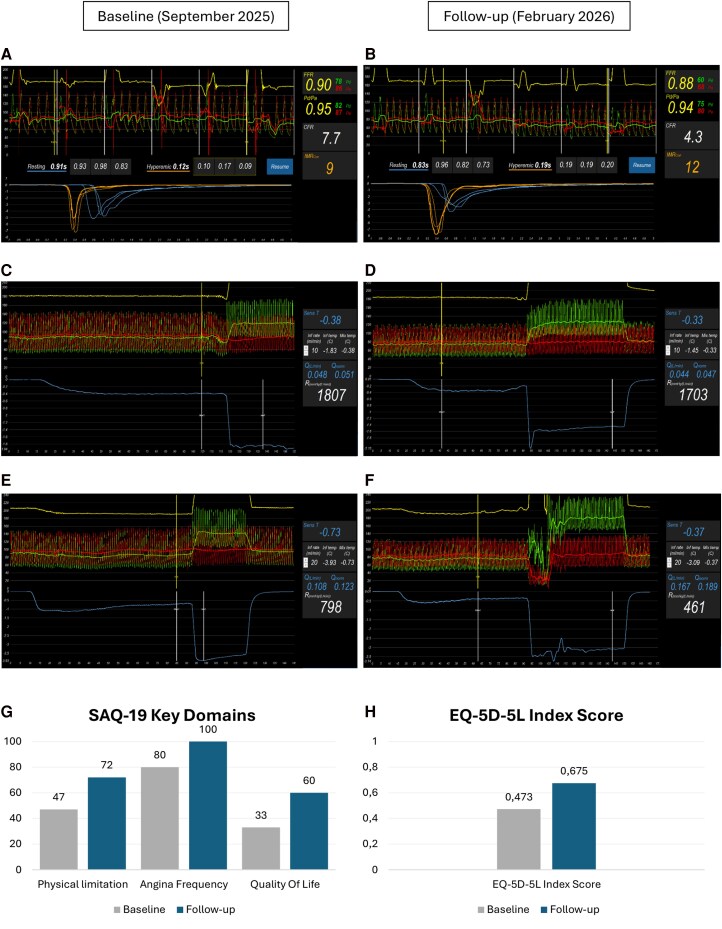
Serial invasive and clinical assessment before and after guideline-directed therapy. *(A–B)* Bolus thermodilution measurements. Baseline derived CFR 7.7 and IMR 9; follow-up derived CFR 4.3 and IMR 12. *(C–F)* Continuous thermodilution measurements at rest and during hyperaemia. Baseline CFR 2.2 and minimal microvascular resistance 798 WU; follow-up CFR 3.8 and minimal microvascular resistance 461 WU. *(G)* Seattle Angina Questionnaire (SAQ-19) key domains demonstrating improvement in physical limitation (47–72), angina frequency (80–100), and quality of life (33–60). *(H)* EQ-5D-5L index score showing improvement from 0.473 at baseline to 0.675 at follow-up.

## Case presentation

### Baseline clinical assessment

An 82-year-old woman with hypertension, hypercholesterolaemia, chronic kidney disease, hypothyroidism, and a previous smoking history (50 pack-years) presented with typical angina worsening over two years, accompanied by exertional dyspnoea (New York Heart Association class II). Physical examination was unremarkable, with normal cardiovascular findings and no signs of heart failure. Myocardial scintigraphy demonstrated moderate reversible stress-induced ischaemia in the apical and lateral territories. Transthoracic echocardiography showed preserved left ventricular ejection fraction 66% without regional wall motion abnormalities.

### Initial invasive coronary evaluation (September 2025)

Coronary angiography revealed non-obstructive coronary arteries with a proximal left anterior descending (LAD) artery stenosis <50%. The fractional flow reserve (FFR) measured at 0.90, excluding functionally significant epicardial disease. At the time of the initial invasive assessment, blood pressure was 144/78 mmHg, heart rate 64 bpm, and rate-pressure product 9216. Haemoglobin was 118 g/l and creatinine level was 97 µmol/l.

Invasive microvascular assessment was performed in the mid-LAD using a pressure–temperature sensor guidewire (PressureWire X, Abbott) and continuous thermodilution through a dedicated monorail infusion catheter (RayFlow, Hexacath). Hyperaemia was induced using continuous intracoronary saline infusion at 20 ml/min according to the standard continuous thermodilution protocol. Intracoronary nitrates were systematically administered before physiological assessment. Antianginal medications and caffeine were withheld prior to both procedures whenever feasible. Coronary flow reserve was 2.2, and minimal microvascular resistance was significantly increased, measuring 798 WU, fulfilling the predefined study criteria for structural CMD based on CFR <2.5 and minimal microvascular resistance >500 WU (*Summary Figure*, *C* and *E*). Parallel assessment using bolus thermodilution yielded markedly discordant results (derived CFR 7.7; IMR 9), values within ranges typically considered normal (*Summary Figure*, *A*). Measurements were repeated within each session in order to minimize variability. Identical guidewire positioning during follow-up assessment was ensured using both angiographic landmarks and wire positioning, with the distal pressure wire sensors positioned at the level of an easily identifiable septal branch in the distal LAD. In the context of consistent clinical findings, objective stress-induced ischaemia, and predefined study criteria prioritizing absolute flow quantification, the diagnosis of INOCA with structural CMD was adjudicated.

### Sequential pharmacological optimization (October–November 2025)

A structured, symptom-guided pharmacological optimization strategy was initiated on 1 October 2025.

Background therapy was initiated with the once-a-day administration of perindopril 5 mg and rosuvastatin 20 mg. Nebivolol 2.5 mg daily was introduced as first-line antianginal therapy. Within one week (8 October 2025), symptomatic bradycardia occurred (heart rate 50 bpm), prompting the reduction of nebivolol to 1.25 mg and up-titration of perindopril to 7.5 mg. Perindopril was further increased to 10 mg on 21 October 2025 as anginal symptoms persisted. Due to recurrent bradycardia (heart rate 45 bpm), nebivolol was eventually discontinued on 24 October 2025.

Given the intolerance to beta-blockade, amlodipine 5 mg daily was initiated on 29 October 2025 and up-titrated to 10 mg daily on 6 November 2025, resulting in a progressive reduction in anginal frequency. However, due to residual weekly symptoms, ranolazine was introduced on 12 November 2025 at 375 mg twice daily and increased to 500 mg twice daily on 26 November 2025. This final combination of treatments was well tolerated haemodynamically with no significant side effects reported by the patient.

### Follow-up clinical assessment (January 2026)

By early January 2026, the patient reported near-complete resolution of typical angina, with only rare, brief, non-limiting episodes of chest discomfort. Her functional capacity also improved substantially, with walking duration increased from approximately 20 min at baseline to 45 min without limitation. LDL cholesterol approached target levels (1.52 mmol/l on 24 October 2025).

Patient-reported outcome measures demonstrated parallel improvement. Seattle Angina Questionnaire scores increased in the domains of physical limitation (47–72), angina frequency (80–100), and quality of life (33–60). The EQ-5D-5L index improved from 0.473 to 0.675 (*Summary Figure*, *G* and *H*). EQ-5D-5L index values were calculated using the England EQ-5D-5L value set. The EQ-5D-5L questionnaire was completed by the patient in the presence of the physician, with assistance provided when clarification was required.

### Follow-up invasive reassessment (February 2026)

The repeat invasive physiological assessment was performed within the framework of the prospective STAR-INOCA research study evaluating the longitudinal evolution of coronary microvascular physiology under structured medical therapy, for which the study design and rationale have been published previously.^11^ The study protocol was approved by the local ethics committee (CER-VD approval number 2025-00306), and the patient provided written informed consent for study participation and repeat invasive assessment. At follow-up assessment, blood pressure was 137/78 mmHg, heart rate 66 bpm, and rate-pressure product 9042. Haemoglobin was 121 g/l and creatinine level was 119 µmol/l. A repeat physiological assessment in the LAD using the same continuous thermodilution protocol and identical guidewire position demonstrated a marked improvement in microvascular function, with CFR increasing by 73% to 3.8, and minimal microvascular resistance decreasing by 42% to 461 WU (*Summary Figure*, *D* and *F*). Repeat measurements were performed under the same procedural conditions and physiological protocol. This corresponded to an increase in absolute hyperaemic coronary flow of 54.6%. FFR changed minimally from 0.90 to 0.88, remaining clearly above the ischaemic threshold. Repeat bolus thermodilution measurements at follow-up showed a derived CFR of 4.3 and an IMR of 12 (*Summary Figure*, *B*). Of note, there were no procedural complications.

**Figure 1:**
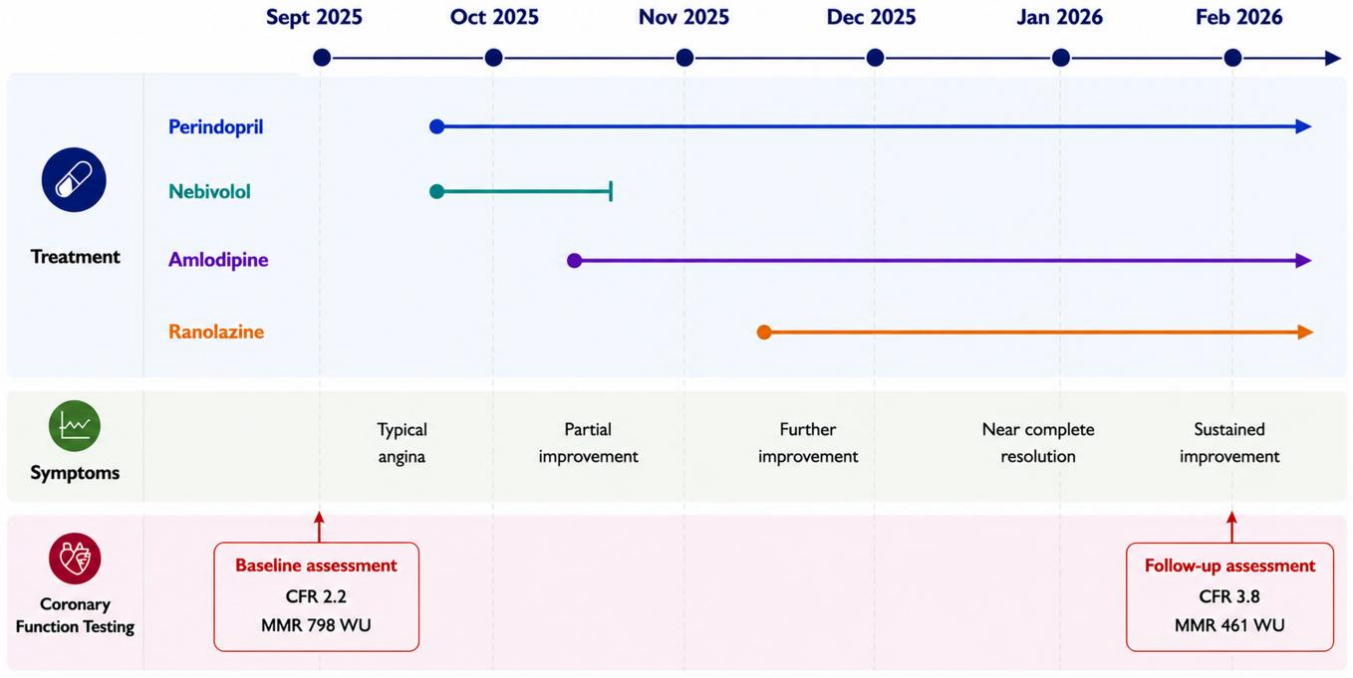
Structured timeline illustrating sequential pharmacological interventions, symptom evolution, and serial invasive coronary function assessments over follow-up. CFR, coronary flow reserve; MMR, minimal microvascular resistance.

A structured overview of treatment modifications, symptom evolution, and serial invasive coronary function assessments throughout follow-up is provided in *Figure 1*.

## Discussion

This case provides serial invasive assessment demonstrating changes in coronary microvascular physiology following structured pharmacological optimization in a patient with structural CMD, as illustrated in the Summary Figure. The change in CFR (2.2–3.8) and minimal microvascular resistance (798–461 WU) occurred without meaningful epicardial modification (FFR changed minimally from 0.90 to 0.88, in the context of increased hyperaemic flow following reduction in microvascular resistance).

Whilst a highly reproducible technique such as continuous thermodilution exhibits a degree of inter-measurement variability,^4,5^ the magnitude of the improvement in physiological indices in the present case, along with the strongly concordant reduction in symptoms, is consistent with a genuine improvement in microvascular function.

The observed physiological changes may reflect the cumulative effects of therapies targeting complementary pathways. ACE inhibition may enhance endothelial signalling and vasodilatory reserve, calcium-channel blockade reduces microvascular tone and facilitates hyperaemic flow, and ranolazine exerts metabolic anti-ischaemic effects without major haemodynamic impact. Finally, intensive lipid lowering may contribute to endothelial stabilization over time.^1,3^ However, causality cannot be established from a single case involving multiple simultaneous and sequential therapeutic interventions. Several alternative or contributory explanations should therefore be considered, including physiological and haemodynamic variability between procedures, regression to the mean, natural variability in symptoms and physiology, placebo effects, and measurement variability. In addition, intensive lipid lowering may itself have contributed to endothelial improvement over time. Smoking cessation and changes in physical activity may also potentially influence coronary microvascular physiology and symptom perception. Interpretation of continuous thermodilution-derived indices also requires caution, as currently available reference values remain relatively broad. In addition, absolute coronary blood flow and microvascular resistance are influenced by the myocardial mass subtended by the interrogated coronary territory, which may partly contribute to the variability observed across published reference values. While structural CMD has often been conceptualized as reflecting relatively fixed microvascular abnormalities, the present case suggests that functional and dynamic components may coexist that are modifiable under sustained therapy.

These observations have practical implications. First, structured, guideline-directed medical therapy as recommended by contemporary consensus documents^1^ may be associated not only with symptomatic benefit but also with a measurable improvement in invasive microvascular physiology. Second, when absolute coronary flow has been quantified at baseline, repeat invasive assessment performed in selected research settings may provide objective documentation of therapeutic response in selected patients. Continuous thermodilution, by directly measuring absolute flow and resistance, appears particularly suited for longitudinal intra-patient follow-up.^4^

At present, however, invasive coronary function testing remains primarily a diagnostic and stratification tool rather than a real-time physiology-guided platform for individualized pharmacological selection.

A notable finding was the baseline discordance between bolus and continuous thermodilution-derived indices, with normal IMR and derived CFR values despite reduced absolute coronary flow and elevated minimal microvascular resistance. Such discordance has been previously reported and should be interpreted within an integrated diagnostic framework, as emphasized by the EAPCI consensus, while also reflecting important methodological differences between the two techniques rather than necessarily indicating technical error.^1,5^ Bolus thermodilution relies on transit-time measurements that are sensitive to injection technique and haemodynamic conditions and may become less reliable when transit times approach lower physiological limits. In contrast, continuous thermodilution enables direct quantification of absolute coronary blood flow and minimal microvascular resistance under steady-state hyperaemia.^4,7^

In particular, Gallinoro *et al*.^5^ demonstrated that continuous thermodilution yields lower CFR and MRR values compared with bolus thermodilution, accompanied by an almost three-fold reduction in measurement variability in patients with ANOCA. More recently, Mahendiran *et al*.^6^ further supported the reproducibility and methodological robustness of continuous thermodilution assessment using simplified infusion protocols.

While technical factors can never be completely excluded, all measurements in the present case were performed using a standardized protocol by an experienced coronary physiology team. Importantly, measurements were reproducible within each session and were interpreted in the context of concordant clinical presentation and objective stress-induced ischaemia, supporting the overall physiological interpretation.

Several limitations warrant consideration. First, thermodilution-derived indices remain subject to physiological and procedural variability. However, the use of an identical invasive protocol and the magnitude of measured change reduce the likelihood that findings are attributable solely to measurement variability. Second, functional indices cannot directly assess structural remodelling; whether the observed improvement reflects reversal of structural abnormalities or modulation of superimposed functional impairment remains uncertain. In addition, although invasive coronary physiology may contribute to therapeutic stratification in INOCA, the present protocol was not designed to evaluate acute physiology-guided pharmacological selection based on immediate invasive responses to different therapies. Future personalized approaches involving sequential intracoronary testing of therapeutic agents may be conceivable but currently remain limited by important practical and methodological constraints, including prolonged procedural duration, the need for repeated thermodilution measurements, and potential carry-over effects between sequentially administered therapies. Acetylcholine testing was not performed in the present case. Although the exclusively exertional symptom pattern made a predominantly vasospastic phenotype appear less likely, the absence of rest angina cannot reliably exclude vasospastic or mixed endotypes. Accordingly, mixed pathophysiological mechanisms may still have contributed to the observed clinical and physiological findings. Recent data from our group suggest that selected clinical characteristics may help identify patients more likely to benefit from vasospasm testing. Finally, this report describes a single patient and thus cannot establish generalisability.

In summary, this case illustrates that structural CMD may exhibit measurable physiological improvement under sustained, guideline-directed therapy and highlights the value of quantitative absolute coronary flow assessment for documenting microvascular adaptation over time.

## Data Availability

The data underlying this article will be shared on reasonable request to the corresponding author.

## Lead author biography

Jérémie Buri graduated from the University of Lausanne in 2022 and completed two years of training in internal medicine followed by six months in intensive care. He is currently an MD candidate and cardiology resident at the University Hospital in Lausanne (CHUV), focusing on coronary physiology and microvascular dysfunction, with a particular interest in invasive coronary function testing and INOCA.

## Notes

**Consent:** The authors confirm that written consent for submission and publication of this case report, including images and associated text, has been obtained from the patient, in line with COPE guidance.
